# Shufeng Jiedu capsule alleviates influenza A (H1N1) virus induced acute lung injury by regulating the lung inflammatory microenvironment

**DOI:** 10.1016/j.heliyon.2024.e33237

**Published:** 2024-06-18

**Authors:** Xiaorui Wang, Zihan Geng, Yanyan Bao, Juying Zhong, Jing Ma, Xiaolan Cui, Yujing Shi

**Affiliations:** a Institute of Chinese Materia Medica, China Academy of Chinese Medical Sciences, Beijing, 100025, China; b Experimental Research Center, China Academy of Chinese Medical Sciences, Beijing, 100025, China

**Keywords:** Influenza A virus, Pneumonia, Cellular immune response, Inflammation, Cytokine

## Abstract

**Background:**

Death caused by respiratory tract infection is one of the leading causes of death in the world today. Shufeng Jiedu Capsule (SFJDC) is a traditional Chinese medicine that has been widely used clinically for coronavirus disease 2019 (COVID-19), H1N1 influenza virus pneumonia and other diseases. Its pharmacological effect is to inhibit inflammation and improve the body's ability to clear viruses. However, the mechanism of SFJDC in the treatment of viral pneumonia, especially its effect on the inflammatory-immune microenvironment of lung tissue remains unclear.

**Methods:**

Mice with H1N1 influenza virus pneumonia were used as a model to verify the efficacy of SFJDC through death protection, lung index, viral load, and HE staining of lung tissue. The levels of inflammatory cytokines and chemokines in lung tissue were investigated by multi-analyte immunoassay. The number and proportion of cells in peripheral blood were detected by blood routine. The percentage of infiltrating immune cells in lung tissue was detected by flow cytometry and immunofluorescence.

**Results:**

SFJDC (2.2 g/kg·d^−1^ and 1.1 g/kg·d^−1^) increased survival rate (*P*＜0.01, *P*＜0.05), prolonged the survival period of mice, and alleviated the histopathological damage in lung (*P*＜0.01). SFJDC (2.2 g/kg·d^−1^, 1.1 g/kg·d^−1^ and 0.055 g/kg·d^−1^) increased body weight(*P*＜0.01, *P*＜0.05), improved activity status, reduced the lung index (*P*＜0.01, *P*＜0.05) and viral load (*P*＜0.01). SFJDC (2.2 g/kg·d^−1^ and 1.1 g/kg·d^−1^) reduced interleukin-1β (IL-1β), interleukin-18(IL-18), tumour necrosis factor α (TNF-α), monocyte chemoattractant protein (MCP), chemokine (C-X-C motif) ligand 1 (CXCL1) (*P*＜0.01, *P*＜0.05), and SFJDC (2.2 g/kg·d^−1^) increased IL-10 levels (*P*＜0.05) to regulate inflammation. SFJDC (2.2 g/kg·d^−1^) increased the percentages of CD4^+^ T cells (*P*＜0.01), CD8^+^ T cells (*P*＜0.05), and B cells(*P*＜0.05), and decreased F4/80^+^ macrophages (*P*＜0.05).

**Conclusion:**

Our findings indicated that SFJDC could inhibit inflammation and lung injury while maintaining the function of the adaptive immune response mediated by T and B cells, and promote the clearance of the virus, thereby treating influenza A (H1N1) virus-induced pneumonia.

## Highlights

• SFJDC has positive effect on the lung inflammatory microenvironment.
• SFJDC maintains the function of the adaptive immune response mediated by T and B cells.
• The results show SFJDC alleviates influenza A (H1N1) virus induced acute lung injury at the early stage.

## 1 Introduction

Influenza virus causes more than one billion infections and hundreds of thousands of deaths worldwide each year [1]. H1N1 is a subtype of type A influenza viruses. Its infection initiates by binding to the sialylated protein on the surface of the respiratory tract and lung epithelial cells [2]. While host immunity responds to kill the virus, it also causes damage to the respiratory tract and lungs, which is manifested as airway inflammation, protein exudation and alveolar necrosis. Clinically, it leads to breathing difficulties, acute respiratory distress syndrome (ARDS) and even death [3].

Shufeng Jiedu Capsule (SFJDC) is a Traditional Chinese Medicine composed of *Polygoni cuspidati rhizoma* (Huzhang), *Forsythiae fructus* (Lianqiao), *Isatidis radix* (Banlangen), *Bupleuri radix* (Chaihu), *Patriniae herba* (Baijiangcao), *Verbenae herba* (Mabiancao), *Phragmitis rhizoma* (Lugen), *Glycyrrhizae radix* (Gancao), in which 41 chemical constituents of SFJDC were identified using the ultra-high performance liquid chromatography-quadrupole time-of-flight mass spectrometry (UPLC/Q-TOF-MS) [4]. Many studies have confirmed that SFJDC has curative effect in combination with western medicine in the clinical treatment of pneumonia caused by respiratory infections such as influenza A virus and Severe Acute Respiratory Syndrome Coronavirus 2 (SARS-CoV-2) [5,6]. It has anti-inflammatory and immunomodulatory effects in various acute lung injury models *in vivo* [[7], [8], [9]]. Mechanism studies have shown that the effective constituents of SFJDC can inhibit activation of macrophages through Mitogen-activated protein kinase (MAPK)/Nuclear factor kappa-B (NF-κB) pathway, reduce inflammatory cytokines such as interleukin-1β (IL-1β) and tumour necrosis factor α (TNF-α) in lung tissue [8,10], thereby regulating the inflammatory response.

Inflammatory cytokines are mainly produced by immune cells such as neutrophils and macrophages, which can activate T and B cells to initiate antiviral adaptive immune response [11,12]. However, excessive inflammation triggers cytokine storms and leads to death [13,14]. At the same time, excessive inflammatory response will lead to inhibition of lymphocytes, reducing the body's antiviral ability. Therefore, inhibiting inflammation while protecting the body's antiviral immune response are important in the treatment of pneumonia caused by viral infection. However, the effect of SFJDC on the proportion of lung-infiltrating immune cells is still unclear.

In this study, mice model of pneumonia induced by influenza A virus (H1N1) strain A/PR/8/34 were used to investigate the cytokine levels and immune cell proportions in the inflammatory microenvironment of peripheral blood and lung tissue. The possible mechanism of SFJDC in relieving inflammation and protecting adaptive immune response in the early stage of pneumonia was discussed.

## 2 Materials and methods

### 2.1 Mice

Male and female BALB/c mice (5-week-old, 14 ± 1 g) were purchased from Beijing Vital River Laboratory Animal Technology Company. All mice were housed in a pathogen-free facility at the ABSL-2 laboratory of Institute of Chinese Materia Medica, China Academy of Chinese Medical Sciences.

### 2.2 Virus preparation

Influenza A virus (H1N1) strain A/PR/8/34 (PR8) (VR-95™, ATCC), were passaged in chicken embryos and stored at −80 °C. The virus titer was determined by the chicken hemagglutination test, and the median lethal dose (LD_50_) was determined. The relevant experiments were all carried out in animal biosafety level 2 (ABSL-2) laboratory.

### 2.3 Establishment of mice pneumonia model

Mice were anesthetized with isoflurane (EZVET), and PR8 virus diluted with normal saline was infected at a volume of 35 μl/mouse intranasally. The day of infection was defined as day 0, the autopsy was performed on day 4 and the endpoint of death protection observation was day 14. In the death protection and other experiments, BALB/c mice were infected with intranasal drops using 5 LD_50_. Bodyweights were collected on day 0 and day 4. The animals were euthanized on the 4th day of the experiment using CO_2_ asphyxiation. Lung tissue and peripheral blood samples were collected during the procedure. Mice were observed for activity status every day from day 0 to day 14. On the 14th day of the death protection experiment, euthanasia was performed using CO_2_ asphyxiation.

### 2.4 Drug preparation and administration

SFJDC (Anhui Jiren Pharmaceutical, 3190923) was dissolved in distilled water to 2.2 g/kg·d^−1-^(equivalent to raw drug dosage of 11.42 g/kg·d-1), 1.1 g/kg·d^−1^ and 0.055 g/kg·d^−1^ corresponding to double, equal and 1/2 times of clinical dosage. Oseltamivir phosphate (Roche Pharmaceutical, M1069) was dissolved to 0.083 g/kg·d^−1^. All drugs were intragastrically administered at 20 ml/kg·d^−1^ once a day on the 0–3 day of infection.

### 2.5 RNA isolation and quantitative RT-PCR

Total RNA of lung tissue was extracted using TRIzol™ (Thermo Fisher Scientific, 15596026). One Step TB Green™ PrimeScript™ RT-PCR Kit (Takara, RR086A) was used for RT-qPCR according to the manufacturer's protocol. Reverse transcription was performed at 42 °C for 5 min, followed by pre-denature at 95 °C for 10 s. Then denaturing at 95 °C for 5 s, annealing and extension at 60 °C for 34s were repeated for 40 cycles. PCR amplification was analyzed by 2^−ΔΔCT^ method using SDS v1.4 design and analysis software (Applied Biosystems, 7500 Real Time PCR System).

GAPDH: Sense primer: GGAGAGTGTTTCCTCGTCCC; and anti-sense primer: ATGAAGGGGTCGTTGATGGC.

Influenza A virus: Sense primer: CGAAGTGGGAGCCAGGATAC; and anti-sense primer: ATCTCGTTTTGCGGACCAGT.

### 2.6 Peripheral blood cell detection

The peripheral blood of the mice was added into EDTA anticoagulant tubes, then hemocytes were detected using Sysmex XN-550 hematology analyzer.

### 2.7 Hematoxylin-eosin (H&E) and pathological scoring

Lung fixed in formalin were dehydrated with a gradient of alcohol, soaked in xylene, and then embedded in paraffin. The embedded wax block was cut into 4–6 μm slices, then flattened lightly on glass slides and dried in a thermostat. The slides were stained by H&E staining, and the pathological changes of the lung tissue were observed under an optical microscope and photographed.

The histopathology score was given by the following rules. No inflammatory exudation, edema, or obvious blood stasis = 0, mild exudative inflammation in the lung interstitium, no obvious inflammation in or around the bronchioles of the lung = 1, limited exudative inflammation and small hemorrhagic staining in lung interstitium, slight inflammatory exudation around the bronchioles = 2, hemorrhagic reddening in lung interstitium, accompanied by alveolar collapse, exudative inflammation and cell fragmentation in lung tissue = 3.

### 2.8 Cytokine and chemokine detection

50 mg of mouse lung tissue was placed in 2 mL RIPA lysate, the total protein of the lung tissue was extracted, and the protein concentration was determined. The samples were uniformly diluted with phosphate buffered saline (PBS) to a protein concentration of 10 mg/ml. The high-throughput liquid-phase protein chip detection using ProcartaPlex Mix&Match Mouse 9-plex (Invitrogen, EPX480-20834-901) was carried out according to the manufacturer's protocol, and the protein concentration was detected using Luminex 200 liquid suspension chip system.

### 2.9 Lung tissue dissociation

Lung tissues were placed in C tubes (Miltenyi Biotec, 130-093-237) with digestive enzymes prepared according to the instructions of Mouse Lung Dissociation Kit (Miltenyi Biotec, 130-100-008), and dissociated using the 37C_m_LDK_1 program of GentleMACS Octo (Miltenyi Biotec). While awaiting dissociation, lungs were placed in MACS Tissue Storage Solution (Miltenyi Biotec, 130-100-008). The dissociated cell suspension was then filtered and centrifuged for subsequent FACS staining.

### 2.10 Flow cytometry

Before staining, cells were blocked with 5 % FBS in PBS. Cells were stained with the following fluorophore-conjugated antibodies purchased from Invitrogen: APC-eFluor™ 780 labeled Anti-Mouse CD45 (47-0451-82), Alexa Fluor 488 labeled Anti-CD170 (Siglec-F) Monoclonal Antibody (53-1702-82), PE labeled Anti-Mouse CD64 (X54-5/7.1) (12-0641-82). The following fluorophore-conjugated antibodies were purchased from TONBO biosciences: FITC labeled Anti-Mouse CD3e (35-0031), PerCP-Cyanine5.5 labeled Anti-Mouse CD4 (25–0041), APC labeled Anti-Mouse CD8a (65–0081), APC labeled Anti-Mouse CD45 (20–0451), PerCP-Cyanine5.5 labeled Anti-Human/Mouse CD11b (65–0112), PE-Cyanine7 labeled Anti-Mouse CD11c (60–0114), FITC labeled Anti-Mouse F4/80 (35–4801), PE labeled Anti-Mouse Ly-6G (50–5931), PE labeled Anti-Mouse CD19 (50–0193). After washing with PBS and resuspending in 2 % paraformaldehyde, cells were collected on the FACSCelesta flow cytometer (BD Biosciences) and analyzed using FlowJo software.

### 2.11 Multiplex immunohistochemistry (IHC) staining

Multiplex IHC staining was performed on 4-μm-thick, formalin-fixed, paraffin-embedded slides using an Opal multiplex IHC system (NEL811001KT, PerkinElmer) according to the manufacturer's instructions. After heat-induced epitope retrieval, slides were blocked with PerkinElmer Antibody Diluent Block buffer, and incubated with the following primary antibodies: Anti-CD20 antibody (Abcam, Ab64088), F4/80 (D2S9R) XP® Rabbit mAb (CST, 25514), Anti-CD4 antibody (Abcam, Ab183685), Anti-CD8 antibody (Abcam, Ab217344), Anti-CD19 antibody (Abcam, Ab245235). After washed in TBST, the slides were incubated with Opal polymer HRP Ms + Rb secondary antibody (PerkinElmer, ARH1001EA) and Opal fluorophores (Akoyabio, FP1487001KT, FP1494001KT, FP1488001KT, FP1495001KT, FP1501001KT). After staining with DAPI (Akoyabio, FP1490), images were acquired using the Vectra multispectral imaging system and analyzed by inForm 2.8.0 software (PerkinElmer).

### 2.12 Statistics

All data were analyzed with GraphPad Prism software and presented as a mean ± s.d. Statistical significance was determined by the two-tailed paired or unpaired Student's *t*-test, or one-way analysis of variance (ANOVA) followed by Tukey's multiple-comparisons test, with significance level of *P < 0.05 and **P < 0.01. Nonparametric test was used for data with heterogeneity of variances, and Log-rank (Mantel-Cox) test was used in the survival analysis.

Lung index was calculated as lung wet mass × 100/body weight. Death protection rate was calculated as 100 % × (the number of death in the model group - the number of death in the drug administration group)/the number of deaths in the model group. The calculation method for life extension rate was 100 % × (average survival days of drug administration group - average survival days of model group)/average survival days of model group.

## 3 Results

### 3.1 SFJDC exhibited therapeutic effects on survival protection, lung index and viral load reduction on mice model infected by PR8

**Fig. 1:**
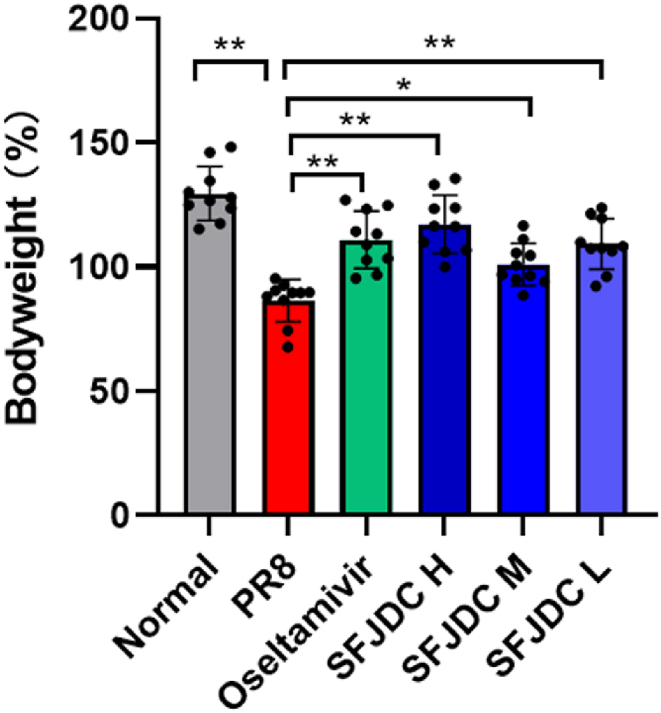
Effects of SFJDC on bodyweight.

During the death protection experiment, we measured the body weight of mice before and after administration. Compared with the normal group, the body weight was significantly decreased after PR8 infection (*P* < 0.01, Fig. 1). Compared with the PR8 group, oseltamivir phosphate and SFJDC significantly increased the body weight (*P* < 0.05, Fig. 1). We also observed the activity status. All Mice infected intranasally with 5 LD50 of PR8 showed signs of disease, starting on day 2 with hunched posture. Out of 20 mice, 18 individuals became lethargy and died on the same day or the following day during the course of infection. The positive drug oseltamivir phosphate and three doses of SFJDC improved the fur condition and decreased mobility of mice (Table 1).

**Table 1:** Activity status of model mice after drug intervention.

Activity status	No./Total (Days after PR8 infection)				
	PR8	Oseltamivir phosphate	SFJDC H	SFJDC M	SFJDC L
Hunched posture	20/20 (2–9)	10/20 (3–8)	12/20 (3–8)	12/20 (3–8)	13/20 (3–9)
lethargy	18/20 (4–9)	3/20 (5–8)	8/20 (5–8)	10/20 (4–8)	13/20 (4–9)
Ruffled fur	18/20 (3–10)	10/20 (3–9)	12/20 (3–9)	12/20 (3–9)	13/20 (3–10)

Bodyweight loss of mice in each group after PR8 infection. Mice were infected with 5 LD50 of PR8 intranasally, and the positive drug oseltamivir phosphate or the test drug SFJDC were administrated. Bodyweight of mice throughout the experiment was calculated as percentage of start weight (*n* = 10).

**Fig. 2:**
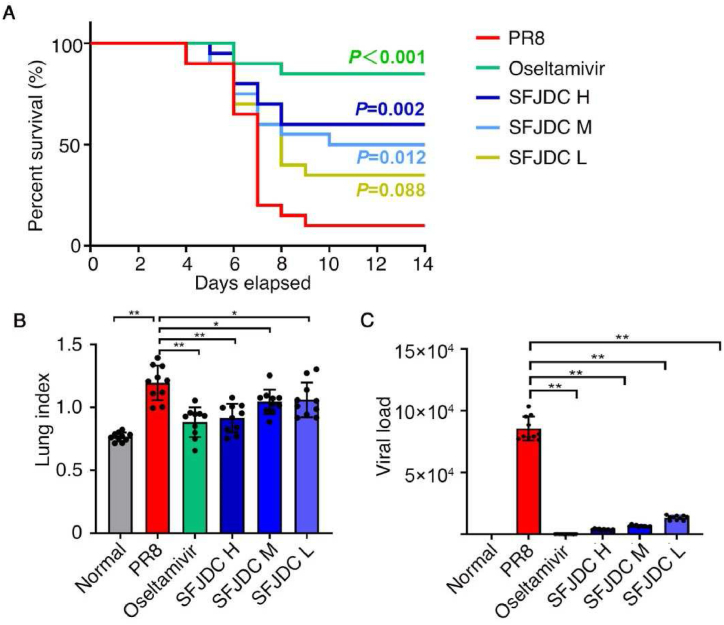
Therapeutic effects of SFJDC. **A.** Survival rate of mice in each group after PR8 infection. Mice were infected with 5 LD_50_ of PR8 intranasally, and the positive drug oseltamivir phosphate or the test drug SFJDC were administrated. Survival rate of the mice is calculated every other day (*n* = 20). **B.** Lung index of mice. Mice were infected with 5 LD_50_ of PR8 intranasally, and administered with oseltamivir phosphate or SFJDC. Uninfected mice were regarded as normal group. The lung index was calculated on the 4th day of infection (*n* = 10). **C.** Viral load of mice. On the 4th day of infection, the whole lung tissue was taken to extract RNA and dissolved with equal volume of DEPC water for viral cDNA amplification (*n* = 10). PR8, mice infected by influenza A virus (H1N1) strain A/PR/8/34 and intragastrically administrated with distilled water. Oseltamivir, mice infected by PR8 and intragastrically administrated with 0.083 g/kg·d^−1^ oseltamivir phosphate. SFJDC H, M and L, mice infected by PR8 and intragastrically administrated with high (2.2 g/kg·d^−1^), medium (1.1 g/kg·d^−1^) and low (0.055 g/kg·d^−1^) dose of SFJDC respectively. *P < 0.05, **P < 0.01.

We evaluated the death-protective effect of SFJDC on model mice. Compared to model group that infected by PR8, the positive drug oseltamivir phosphate and three doses of SFJDC improved the survival rate, death protection rate and life extension rate of mice (Fig. 2A and Table 2). Compared with the normal group, the lung index and lung viral load of PR8-infected mice were significantly increased (*P*＜0.01). Compared with the PR8 group, oseltamivir phosphate, high-dose and medium-dose of SFJDC significantly reduced the lung index and viral load (*P*＜0.01, Fig. 2B and C).

**Table 2:** Death protection and life extension rate of model mice after drug intervention.

	Oseltamivir phosphate	SFJDC H	SFJDC M	SFJDC L
Death protection rate	83.33 %	55.56 %	44.44 %	27.78 %
Life extension rate	75.51 %	50.34 %	40.82 %	27.21 %

### 3.2 SFJDC relieved local inflammation of lung

**Fig. 3:**
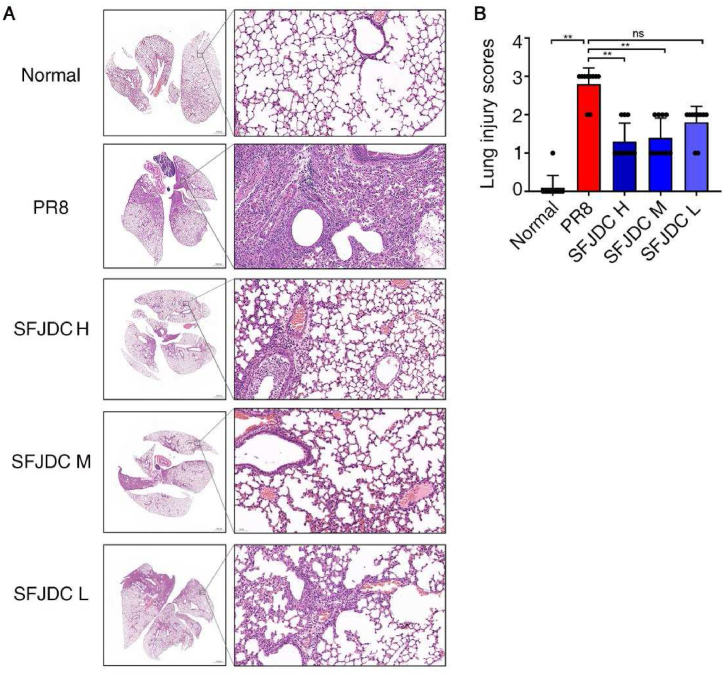
Effect of SFJDC on lung histopathology. **A.** Representative H&E staining of lungs in each group. Mice were intranasally infected with PR8, and intragastrically administrated with SFJDC, or equal volume of distilled water. The uninfected mice were regarded as normal group. On the 4th day of infection, lungs in each group were collected for H&E staining. Scale bar = 1000 μm in the left panel, and scale bar = 50 μm in the right panel. **B.** Corresponding histopathological scores of mice in each group (*n* = 10). *P < 0.05, **P < 0.01.

To determine the anti-inflammatory effect of SFJDC, H&E staining and lung histopathological analysis were performed. Compared with the normal group, we observed thickened alveolar wall, massive inflammatory cell infiltration in the pulmonary alveoli of the PR8-infected mice, significantly elevated histopathological scores. Compared with the PR8 group, high-dose and medium-dose SFJDC significantly relieved lung tissue edema and inflammatory cell infiltration (*P*＜0.01, Fig. 3A and B).

**Fig. 4:**
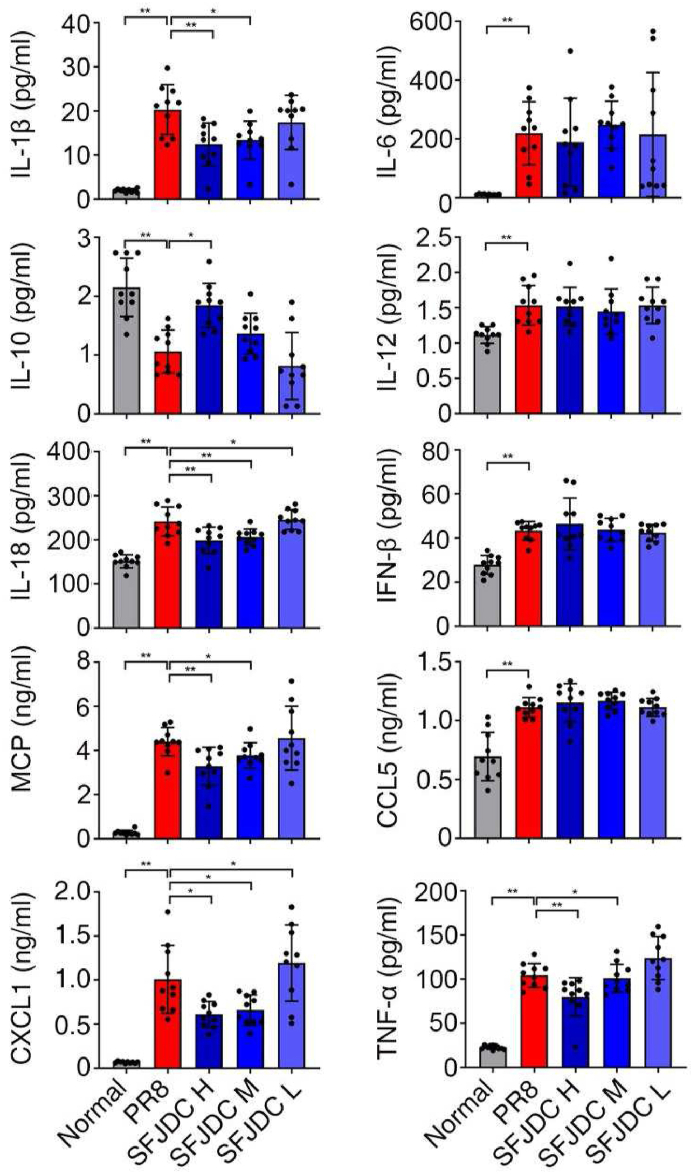
SFJDC reduced the levels of inflammatory cytokines and chemokines in lung tissue. Mice were infected with 5 LD_50_ of PR8 intranasally and administered with SFJDC or equal volume of distilled water. The uninfected mice were used as the normal group. 50 mg of lung tissue was homogenized in 2 ml of PBS for cytokine and chemokine detection on the 4th day of infection (*n* = 10). *P < 0.05, **P < 0.01.

Next, we detected the levels of inflammatory cytokines and chemokines in lung tissues. It was found that compared with the normal group, the inflammatory cytokines IL-1β, IL-6, IL-12, IL-18 and TNF-α in PR8 group were significantly increased, while anti-inflammatory cytokine IL-10 was significantly decreased (*P*＜0.01). Chemokine ligands MCP, CCL5, and CXCL1 were significantly increased (*P*＜0.01). These results indicated that the lungs of model mice had a more obvious inflammatory response. Compared with the PR8 group, IL-1β, IL-18, TNF-α, monocyte chemoattractant protein (MCP) and chemokine (C-X-C motif) ligand 1 (CXCL1) in the SFJDC H group were significantly decreased, and IL-10 was significantly increased. IL-1β, IL-18, TNF-α, MCP, and CXCL1 were significantly decreased in the SFJDC medium group, and CXCL1 was significantly decreased in the SFJDC L group (*P*＜0.05, Fig. 4), suggesting that SFJDC had a significant anti-inflammatory effect.

### 3.3 SFJDC had a protective effect on adaptive immune response in lung

**Fig. 5:**
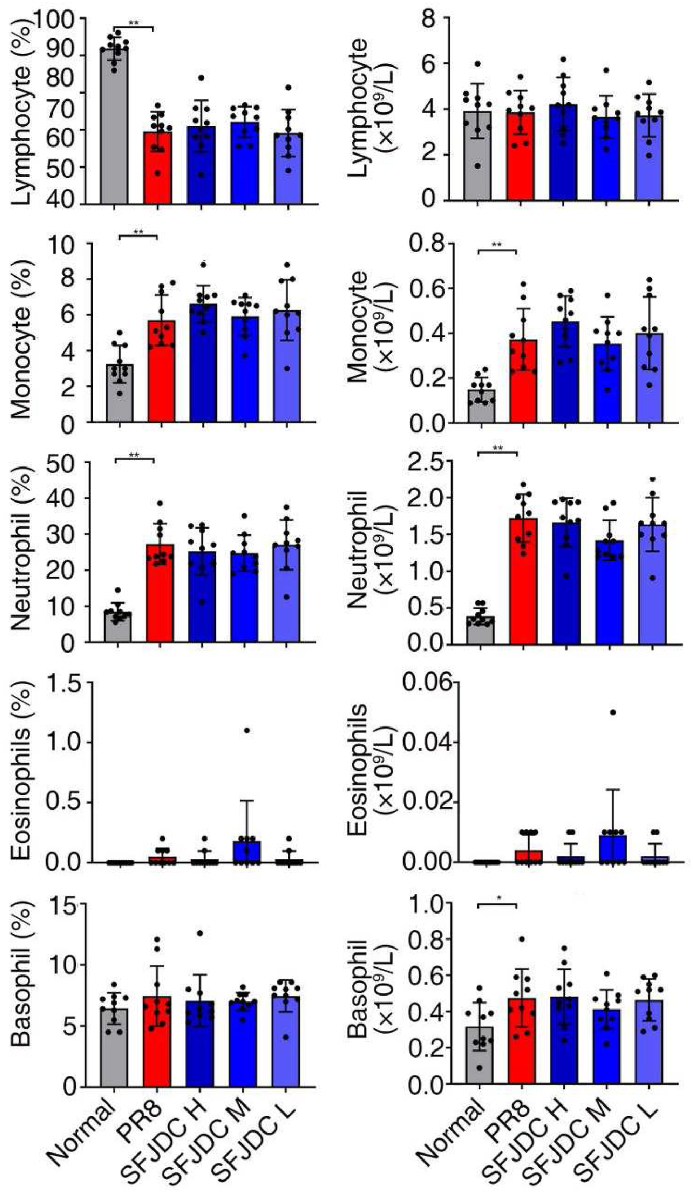
SFJDC had no significant effect on the absolute counts and percentages of peripheral blood cells. Mice were infected by PR8, and administered with SFJDC or equal volume of distilled water. The uninfected mice were used as the normal group. On the 4th day of infection, peripheral blood was collected for blood routine detection (*n* = 10). *P < 0.05, **P < 0.01.

Inflammation is a double-edged sword in the body's defense against viruses. One the one hand, excessive pro-inflammatory cytokines is a crucial cause of cytokine storm and death. On the other hand, moderate inflammatory response is necessary for the recruitment and activation of T and B lymphocytes to play a specific antiviral role. In order to explore whether the reduction of viral load and improvement of survival were due to the anti-inflammatory effect and enhancement of body's antiviral immune response by SFJCD, we first detected the changes of immune cells in peripheral blood. We found that compared with the normal group, the percentage of lymphocytes in PR8-infected mice decreased significantly. The percentage and the absolute count of monocytes and neutrophils, and the number of basophils increased significantly (*P*＜0.01). However, compared with the PR8 group, there was no significant change in neither the percentage nor the absolute count of cells in each group after the administration of SFJDC (Fig. 5).

**Fig. 6:**
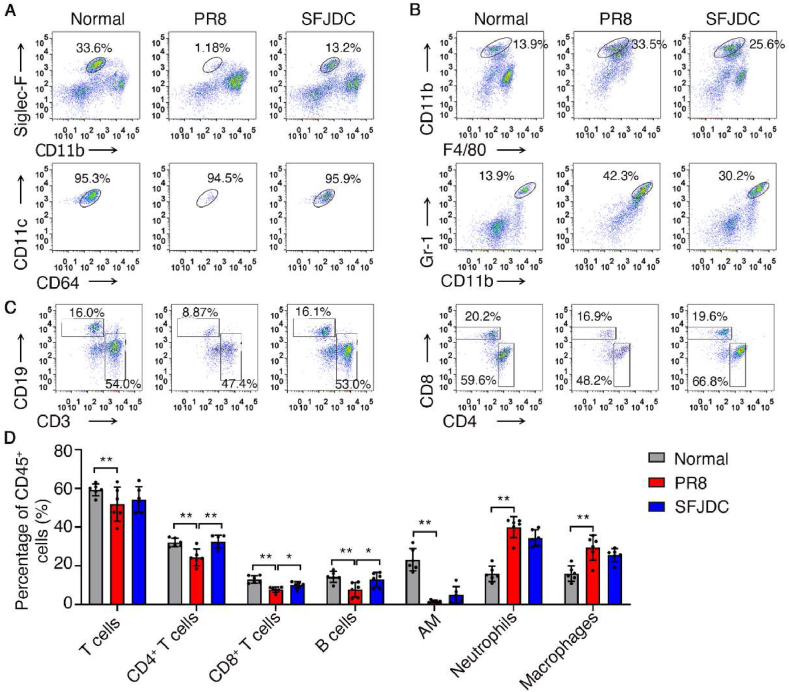
SFJDC reduced the proportion of innate immune cells and increased the proportion of adaptive immune response cells in lung. Mice were infected by PR8 intranasally, and administered with SFJDC (2.2 g/kg·d^−1^) or equal volume of distilled water. The uninfected mice were used as the normal group. On the 4th day of infection, the left lung was collected and dissociated into single cell suspension for flow cytometry detection. **A.** The percentage of Siglec F ^+^ CD11b^dim^CD11c^+^CD64^+^ alveolar macrophages in CD45^+^ cells. **B.** The percentage of CD45^+^CD11b^+^F4/80^int^ monocyte derived macrophages (top) and CD45^+^CD11b^+^Gr-1^+^ neutrophils (bottom) in CD45^+^ cells. **C.** The percentage of CD3^+^ total T cells, CD19^+^ B cells (left), CD3^+^CD4^+^ T cells, CD3^+^CD8^+^ T cells (right) in CD45^+^ cells. **D.** The statistical results of infiltrated immune cell percentage in CD45^+^ cells (*n* = 6). AM, Alveolar Macrophages. *P < 0.05, **P < 0.01.

Given that localized lung tissue could better reflect immune status of pneumonia, we detected the proportions of immune cells infiltrated in lungs. Compared with the normal group, the percentages of CD4^+^ T cells, CD8^+^ T cells, B cells, and alveolar macrophages were significantly decreased after PR8 infection (*P*＜0.01). The percentages of neutrophils and total macrophages were significantly increased (*P*＜0.01). These results suggested acute inflammation of the lungs, the persistence of which can lead to suppression of adaptive immune response. Compared with the PR8 group, high-dose of SFJDC could significantly increase the percentages of CD4^+^ T cells, CD8^+^ T cells, and B cells (*P*＜0.05), but had no significant effect on the percentage of neutrophils, monocyte derived macrophages or alveolar macrophages (Fig. 6).

**Fig. 7:**
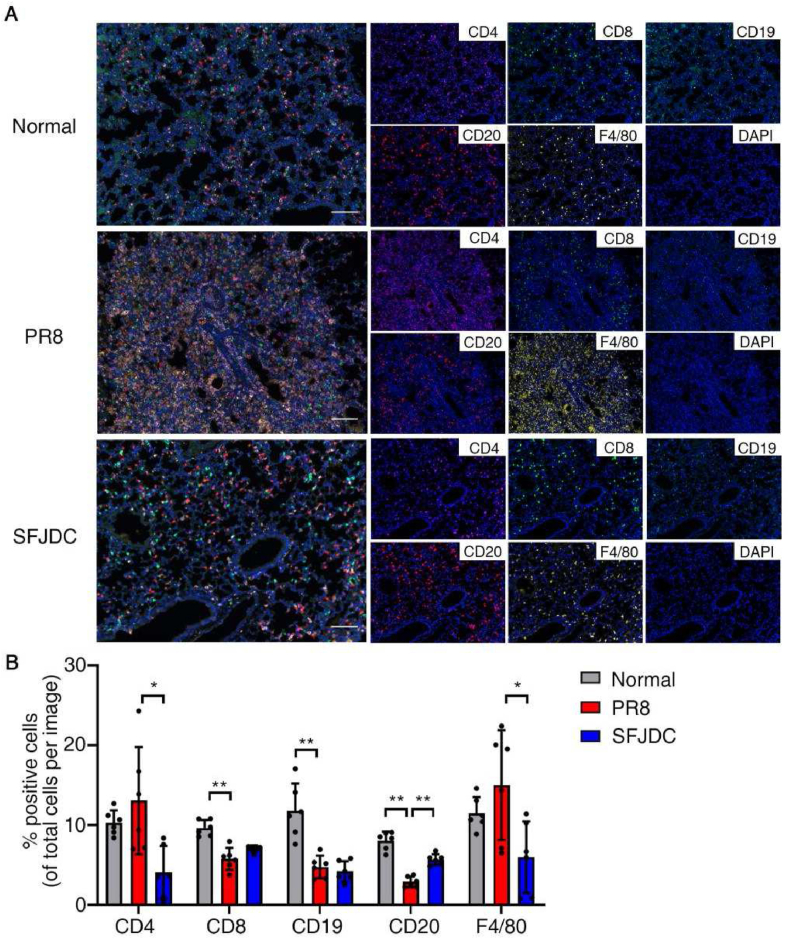
SFJDC reduced the proportion of macrophages in lung. Mice were infected by PR8 intranasally, and administered with SFJDC (2.2 g/kg·d^−1^) or equal volume of distilled water. The uninfected mice were used as the normal group. On the 4th day of infection, the left lungs were collected for multi-color IHC detection. **A.** Overlayed or separately displayed fluorescence imaging of each group. T cells were marked with CD4 and CD8, B cells were marked with CD19 and CD20, and macrophages were marked with F4/80. Scale bar = 100 μm. **B.** Statistics of the immune cell proportions to the total number of cells in the field of view (*n* = 5). *P < 0.05, **P < 0.01.

We also verified the infiltration of immune cells in lung tissue using multi-color IHC staining. Compared with the normal group, the proportion of CD8^+^ T cells, CD19^+^ B cells, and CD20^+^ B cells in the lung tissue of PR8-infected mice were significantly reduced (*P*＜0.01). Although not statistically significant, CD4^+^ T cells, especially F4/80^+^ monocyte-derived macrophages that exacerbate inflammation, tended to increase (*P* > 0.05). After treatment with SFJDC, the proportion of CD20^+^ B cells increased significantly (*P*＜0.01), F4/80^+^ macrophages and CD4^+^ T cells decreased obviously (*P*＜0.05), and the proportion of CD8^+^ T cells tended to increase with no statistical difference (Fig. 7). The above results suggest that SFJDC not only alleviated the inflammation of the lung tissue of PR8-infected mice and improved the survival rate, but may also promote the adaptive immune response, thereby exerting an antiviral effect.

## 4 Discussion

The potential mechanisms of Traditional Chinese medicine in treating viral pneumonia are fascinating, particularly in how it focuses on improving antiviral immunity rather than directly targeting the virus, thereby preventing excessive inflammatory-pathological damage. SFJDC as a traditional Chinese medicine has antipyretic and detoxifying properties, which has been widely used in the treatment of respiratory virus infection. Studies have shown that it alleviated pneumonia and prolonged survival in influenza A virus infection model [7], Lipopolysaccharide (LPS) infection model [8,10,15], *Pseudomonas aeruginosa* infection model [4], mouse model combining disease with syndrome of hCoV-229E pneumonia and cold-dampness syndrome [9], and other acute lung injury models. In this study, we also verified the efficacy of SFJDC in mice with acute lung injury caused by influenza A H1N1 virus, and found that SFJDC can relieve inflammation and reduce viral load in lung. Especially, administration of SFJDC on 0–3 days infection improved the survival of mice for the next 2 weeks, indicating that SFJDC may have potential to prevent the development of viral pneumonia at the early stage [16] of infection. This result is consistent with the idea that Chinese medicine may be particularly effective in treating viral pneumonia in the early stage of infection.

In the context of viral infections, the infected epithelial cells release MCP-1, which recruit neutrophils and monocytes to lung [17]. Together with alveolar macrophages residing in the lung tissue, these cells release type I interferons including IFN-α, IFN-β to directly inhibit virus replication [18]. IL-12, CCL5, CXCL1 were secreted by activated neutrophils, macrophages and other innate immune cells to activate lymphocytes [[19], [20], [21]]. Meanwhile, IL- 1βand IL-18 were produced upon activation of the NLRP3 signaling pathway [22]. Death caused by virus infection is mainly due to rapid virus replication, and uncontrollable inflammation that leads to acute lung injury (ALI) and pulmonary ventilation dysfunction. Uncontrollable inflammation, known as cytokine storm, is triggered by excessive production of pro-inflammatory cytokines such as IL-1β, IL-6, and TNF-α [23,24]. Therefore, we then detected cytokines and chemokines in lung tissue. We found that SFJDC can modulate the expression of these cytokines, including reducing pro-inflammatory cytokines such as IL-1β, IL-18, TNF-α, while increasing the expression of the anti-inflammatory cytokine IL-10. It indicates that SFJDC may play a role in suppressing inflammation and preventing disease exacerbation.

Inflammation is a double-edged sword for the adaptive immune response. On the one hand, moderate inflammation can activate adaptive immune cells such as T and B cells. Upon activation, naïve T cells differentiate into a variety of effector T cells with specific functions [18,25]. CD8^+^ T cells can limit virus replication by secreting interferon, secrete granzyme and other molecules to kill virus-infected lung epithelial cells [18], while CD4^+^ T cells regulate the activation of CD8^+^ T cells and B cells, thereby promoting the production of antibodies and the neutralization of viruses [18,26,27]. On the other hand, high level of inflammatory cytokines and excessive infiltration of neutrophils and macrophages [24] can suppress adaptive immune response, and cause activation-induced cell death (AICD) of lymphocytes, which results in lymphopenia. The exhausted lymphocytes lose their activation, proliferation, differentiation and antiviral ability, and lead to virus evasion [28,29]. Clinical evidences have shown that lymphopenia in peripheral blood is common at the early stage of respiratory virus infection [[29], [30], [31], [32]], which often predicts a poor prognosis and even death [29,33]. Proportion of immune cells infiltrated in lung tissue in our experiments showed that SFJDC reduced the proportion of neutrophils and macrophages, while increasing the proportion of T and B cells. This suggests that SFJDC may work in modulating the immune cell composition within the lung microenvironment, potentially contributing to a more balanced and effective adaptive immune response against viral pneumonia.

In summary, we report here SFJDC has a therapeutic effect against influenza A virus induced acute lung injury, which may be related to the regulation of lung inflammatory microenvironment. However, it has certain limitations. Future studies will compare actions of cytokines and pulmonary pathohistology between oseltamivir (or other antiviral drugs) and SFJDC. We will further clarify the time-course of cytokines and lymphocytes during treatment of infection with SFJDC. Improved understanding of the characteristic impacts of SFJDC on immune regulation may provide new insights into the therapeutic mechanisms and clinical applications of SFJDC.

## 5 Conclusion

Together, our results showed that SFJDC not only modulated immune-inflammatory damage, but also promoted the recovery of T and B lymphocyte ratios, thus protected adaptive immune response that helps the body clear the virus.

## Declarations

### Ethics statement

This study was reviewed and approved by the Institutional Animal Care and Use Committee of Institute of Chinese Materia Medica, China Academy of Chinese Medical Sciences, with the approval number: 2020D42.

## Data availability statement

Part of the data are listed in the supplementary materials. More data will be provide available upon request.

## Funding

This work was supported by the 10.13039/501100012166National Key R&D Program of China (2023YFC2308200 & 2023YFC3502900) and National Key Research and Development Project (grant no. 2018YFE0102300).

## CRediT authorship contribution statement

**Xiaorui Wang:** Writing – review & editing, Writing – original draft, Methodology, Investigation, Formal analysis, Data curation. **Zihan Geng:** Writing – original draft, Project administration, Funding acquisition, Conceptualization. **Yanyan Bao:** Methodology, Investigation, Formal analysis, Data curation. **Juying Zhong:** Project administration, Methodology, Investigation. **Jing Ma:** Investigation, Formal analysis, Data curation. **Xiaolan Cui:** Project administration, Funding acquisition. **Yujing Shi:** Supervision, Resources, Funding acquisition.

## Declaration of competing interest

The authors declare that they have no known competing financial interests or personal relationships that could have appeared to influence the work reported in this paper.

## Figures and Tables

**Table:** 

SFJDC	Shufeng Jiedu Capsule
COVID-19	coronavirus disease 2019
SARS-CoV-2	Severe Acute Respiratory Syndrome Coronavirus 2
ARDS	acute respiratory distress syndrome
MAPK	Mitogen-activated protein kinase
NF-κB	Nuclear factor kappa-B
IL-1β	interleukin-1β
TNF-α	Tumour necrosis factor α
MCP	monocyte chemoattractant protein
CCL5	chemokine (C–C motif) ligand 5
CXCL1	chemokine (C-X-C motif) ligand 1
PR8	Influenza A virus (H1N1) strain A/PR/8/34
LD_50_	median lethal dose
PBS	phosphate buffered saline
LPS	Lipopolysaccharide
ALI	acute lung injury
AICD UPLC/Q-TOF-MS	activation-induced cell death the ultra-high performance liquid chromatography-quadrupole time-of-flight mass spectrometry
